# Novel and Rare Fusion Transcripts Involving Transcription Factors and Tumor Suppressor Genes in Acute Myeloid Leukemia

**DOI:** 10.3390/cancers11121951

**Published:** 2019-12-05

**Authors:** Antonella Padella, Giorgia Simonetti, Giulia Paciello, George Giotopoulos, Carmen Baldazzi, Simona Righi, Martina Ghetti, Anna Stengel, Viviana Guadagnuolo, Rossella De Tommaso, Cristina Papayannidis, Valentina Robustelli, Eugenia Franchini, Andrea Ghelli Luserna di Rorà, Anna Ferrari, Maria Chiara Fontana, Samantha Bruno, Emanuela Ottaviani, Simona Soverini, Clelia Tiziana Storlazzi, Claudia Haferlach, Elena Sabattini, Nicoletta Testoni, Ilaria Iacobucci, Brian J. P. Huntly, Elisa Ficarra, Giovanni Martinelli

**Affiliations:** 1 Department of Experimental, Diagnostic and Speciality Medicine, University of Bologna, 40138 Bologna, Italy; antonella.padella@irst.emr.it (A.P.); simona.righi5@unibo.it (S.R.); guadagnuoloviviana@gmail.com (V.G.); rossella.detommaso@live.it (R.D.T.); cristina.papayannidis@gmail.com (C.P.); valentina.robustell2@unibo.it (V.R.); mariachiara.fontana4@unibo.it (M.C.F.); samantha.bruno2@unibo.it (S.B.); emanuela.ottaviani@aosp.bo.it (E.O.); simona.soverini@unibo.it (S.S.); elena.sabattini@aosp.bo.it (E.S.); nicoletta.testoni@unibo.it (N.T.); 2 Istituto Scientifico Romagnolo per lo Studio e la Cura dei Tumori (IRST) IRCCS, 47014 Meldola (FC), Italy; giorgia.simonetti@irst.emr.it (G.S.); martina.ghetti@irst.emr.it (M.G.); eugenia.franchini@irst.emr.it (E.F.); andrea.ghellilusernadirora@irst.emr.it (A.G.L.d.R.); anna.ferrari@irst.emr.it (A.F.); 3 Department of Control and Computer Engineering DAUIN, Politecnico di Torino, 10129 Turin, Italy; giulia.paciello00@gmail.com (G.P.); elisa.ficarra@polito.it (E.F.); 4 Wellcome Trust-Medical Research Council Cambridge Stem Cell Institute, University of Cambridge, Cambridge CB2 1TN, UK; gg320@cam.ac.uk (G.G.); bjph2@cam.ac.uk (B.J.P.H.); 5 Department of Haematology, Cambridge Institute for Medical Research and Addenbrooke’s Hospital, University of Cambridge, Cambridge CB2 0XY, UK; 6 Institute of Hematology “L. and A. Seràgnoli”, Sant’Orsola-Malpighi University Hospital, 40138 Bologna, Italy; carmen.baldazzi2@unibo.it; 7 MLL-Munich Leukemia Laboratory, 81377 Munich, Germany; anna.stengel@mll.com (A.S.); claudia.haferlach@mll.com (C.H.); 8 Department of Biology, University of Bari Aldo Moro, 70125 Bari, Italy; cleliatiziana.storlazzi@uniba.it; 9 Department of Pathology, St. Jude Children’s Research Hospital, Memphis, TN 38105, USA; ilaria.iacobucci@stjude.org

**Keywords:** acute myeloid leukemia, rare fusion genes, *ZEB2-BCL11B*

## Abstract

Approximately 18% of acute myeloid leukemia (AML) cases express a fusion transcript. However, few fusions are recurrent across AML and the identification of these rare chimeras is of interest to characterize AML patients. Here, we studied the transcriptome of 8 adult AML patients with poorly described chromosomal translocation(s), with the aim of identifying novel and rare fusion transcripts. We integrated RNA-sequencing data with multiple approaches including computational analysis, Sanger sequencing, fluorescence in situ hybridization and in vitro studies to assess the oncogenic potential of the *ZEB2-BCL11B* chimera. We detected 7 different fusions with partner genes involving transcription factors (*OAZ-MAFK*, *ZEB2-BCL11B*), tumor suppressors (*SAV1-GYPB*, *PUF60-TYW1*, *CNOT2-WT1*) and rearrangements associated with the loss of *NF1* (*CPD-PXT1*, *UTP6-CRLF3*). Notably, *ZEB2-BCL11B* rearrangements co-occurred with *FLT3* mutations and were associated with a poorly differentiated or mixed phenotype leukemia. Although the fusion alone did not transform murine c-Kit+ bone marrow cells, 45.4% of 14q32 non-rearranged AML cases were also BCL11B-positive, suggesting a more general and complex mechanism of leukemogenesis associated with BCL11B expression. Overall, by combining different approaches, we described rare fusion events contributing to the complexity of AML and we linked the expression of some chimeras to genomic alterations hitting known genes in AML.

## 1. Introduction

Fusion genes represent a major criterion of diagnosis and prognostic risk stratification in the European Leukemia Net 2017 classification of AML [1], where approximately 18% of cases are characterized by the presence of a known fusion genes as the main driver event [2].

The chromosomal translocation t(15;17), leads to the expression of a *PML-RARA* chimera and characterizes patients with acute promyelocytic leukemia, who generally have favourable prognosis. AMLs expressing the transcripts *RUNX1-RUNXT1* and *CBFβ-MYH11*, associate with the t(8;21) and inv(16), respectively, are also known to confer a favourable prognosis. However, the t(6;9), inv(3)/t(3;3), t(v;11q23.3) and t(9;22) abnormalities result in the expression of *DEK-NUP214*, *GATA2*/*MECOM* fusions, *KMT2A*-fusions and *BCR-ABL1*, respectively, all of which correlate with a poor outcome [1].

Moreover, fusion genes resulting from chromosomal translocations are common features of other haematological cancers and, due to their unique presence in cancer tissues, they represent extremely attractive therapeutic targets. The paradigm is *BCR-ABL1* in chronic myeloid leukemia (CML) and Philadelphia-positive acute lymphoblastic leukemia (ALL), which drives leukemogenesis and can be targeted by a specific therapy capable of reversing the leukemic phenotype [3,4].

Whole genome sequencing and RNA sequencing (RNA-seq) approaches have allowed the identification of several novel fusions in acute leukemia that remained cryptic by routine cytogenetic analysis. The Cancer Genome Atlas Research Network identified 118 fusions in 179 AML patients by RNA-seq, with an average of 1.5 fusions per patient [5]. Moreover, it has been shown that normal karyotype AMLs are characterized by the presence of several chimeras, mainly deriving from adjacent genes located on the same chromosome and with complex patterns of partner gene orientation [6]. Previous studies discovered the *NUP98-PHF23* fusion gene in paediatric cytogenetically normal AML carrying a cryptic chromosomal translocation between chromosomes 11 and 17 [7,8]. Chromosomal translocations leading to the expression of fusion transcripts are also an hallmark of ALL and the detection of such aberrations is an example of how genomic analysis can dramatically improve the sub-classification of patients [9].

Hence, the identification of fusion events, even when shared by a small subgroup of poorly characterized patents, may be of clinical significance. We thus performed RNA-seq on samples from eight AML patients characterized by the presence of a rare or poorly described chromosomal translocation(s) to identify novel fusion transcripts with a potential leukemogenic/pathogenetic role. We also combined different approaches including cytogenetic, RNA-seq, bioinformatics analysis and literature mining to help in understating the pathogenetic role of the identified novel and rare fusion events. We validated the presence of nine fusion genes involving either transcription factors, tumor suppressors, or associated with a loss event of candidate genes in AML. We found that the landscape of alterations in AML is not limited to known genes, and that fusion genes, albeit rare, may play an important role in the disease development.

## 2. Results

### 2.1. RNA-Seq Cohort Selection

We screened the biobank of AML biological samples collected at our Institution between 2010 and 2015. We identified 46 patients (<1% of total cases) carrying a rare chromosomal translocation (i.e., individual incidence <1% [1]) as the sole alteration (13%) or in association with other chromosomal abnormalities (87%). Based on the availability of biological material, eight samples collected at diagnosis or relapse were selected for RNA-seq (Table 1). According to the 2016 revision of WHO classification of myeloid malignancies [10], our cohort included one AML with inv(16)(p13q22) (sample #84), one AML with mutated *NPM1* (sample #63569), one AML without maturation (sample #59810), one AML with maturation (sample #20), one AML with mutated *RUNX1* (sample #21) and three AML cases with myelodysplasia-related changes (samples #32, #68187 and #125). Patients had an average of three mutations per case (range: 1–5). Recurrently mutated genes in our cohort included *DNMT3A* (*n* = 2), *FLT3* (*n* = 3), *IDH2* (*n* = 2), *KDM6A* (*n* = 2) and *TET2* (*n* = 2). All the molecular alterations in myeloid-related genes are listed in Table S2.

### 2.2. Identification and Validation of Fusions Genes

**Figure 1:**
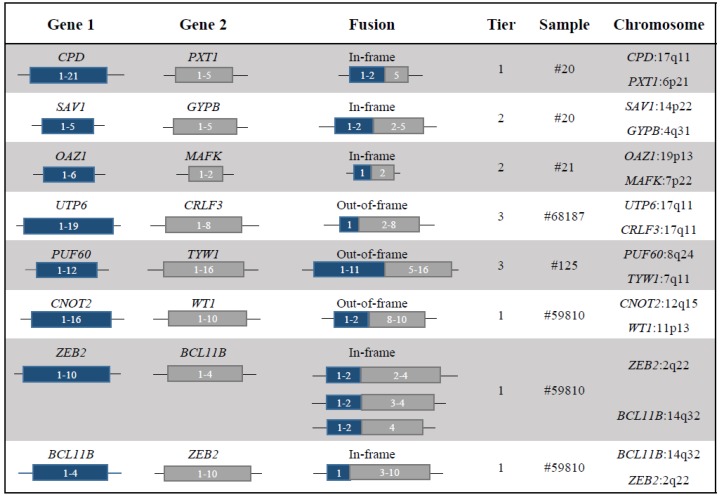
Schematic representation of validated fusion genes. Reading frames, tier, samples in which they were detected and chromosomal location of partner genes are reported. For the *ZEB2-BCL11B* transcript, we detected three splicing isoforms and the reciprocal transcript *BCL11B-ZEB2*.

Among fusions detected by the RNA-seq analysis, we selected 19 for further validation by RT-PCR and Sanger sequencing (Table S3). Of these, 10 were successfully validated, including the known chimera *CBFβ-MYH11* (53% of selected fusions, Figure 1, Table 2 and Table S2). No chimeras were detected and/or confirmed in samples #32 and #63569. The biological information on the putative function of the novel chimeric proteins is described in Table 2. Specifically, a new in-frame fusion gene was identified in sample #20: *CPD-PXT1* [11] (tier 1, Figure 1), which is hypothesized to be the reciprocal fusion product of a t(6;17)(p21;q11) translocation (Figure S1A). *CPD* encodes for a metallocarboxypeptidase and it maps in chromosome 17q11, approximately 625 Kb upstream *NF1*. Copy number analysis from SNParray data revealed that *CPD* had complex rearrangements including a copy number loss of approximately 2 Mb, from chr17:2872554, which maps in the intron 2-3 of *CPD*, to chr17: 30768221, including the entire *NF1* gene (chr17:29419945-29706695, Figure S2A).

**Figure 2:**
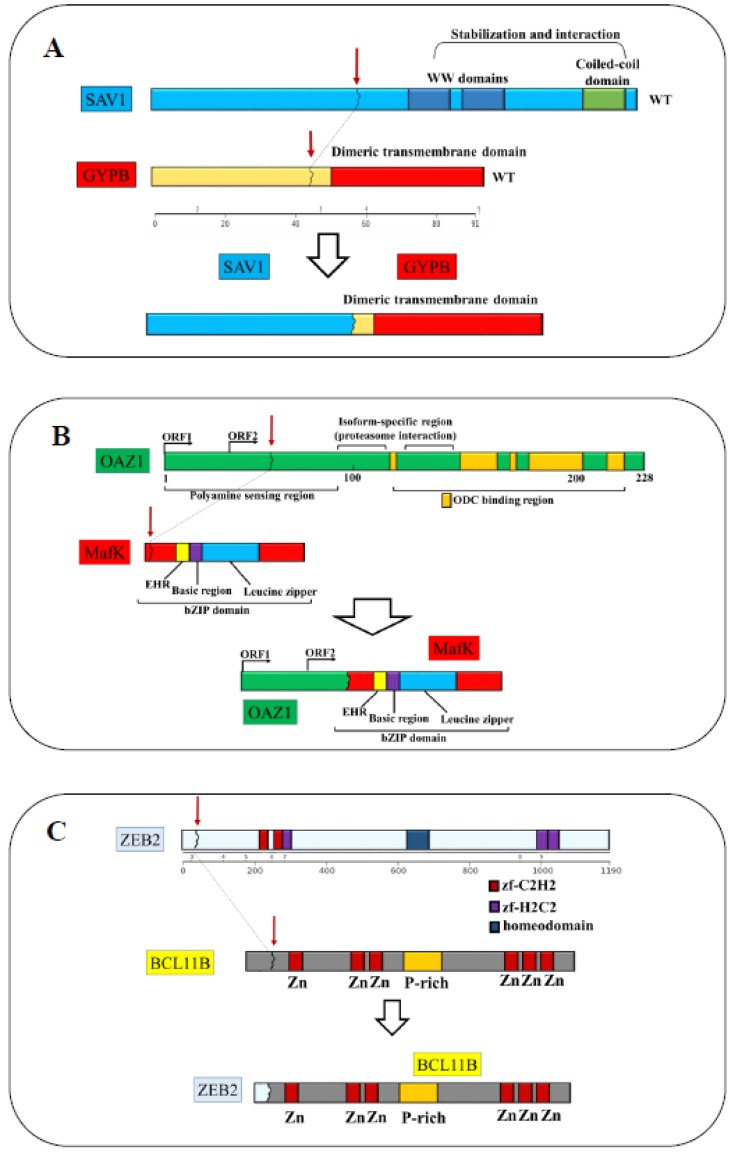
Representation of the domains of the in-frame fusion genes. (**A**) The breakpoint of *SAV1-GYPB* mapped on chromosome 14p22, exon 2 of *SAV1* (NM_021818) and chromosome 4q31, exon 2 of *GYPB* (NM_002100, Figure S1B). In the putative fusion protein, *SAV1* lost the stabilization and interaction domains including the WW domain and the coiled-coil domain, while *GYBP* lost the N-terminal domains and retained the dimeric transmembrane domain. (**B**) The breakpoint of *OAZ1-MAFK* mapped in exon 1 of *OAZ1* (NM_004152), which encodes for a polyamine sensing region and a proteasome interaction domain. The breakpoint at 3’ mapped in exon 2 of *MAFK* (NM_002360), which, together with exon 3, encodes for the bZIP domain. The putative chimeric protein was formed by the sensing regions of polyamine that normally controls the transcription of *OAZ1*, and the bZIP domain of MAFK. (**C**) The breakpoint of the fusion *ZEB2-BCL11B* mapped in exon 2 of *ZEB2* (NM_014795) and exon 2 of *BCL11B* (NM_00128223). Twenty-four residues of ZEB2 and 803 out of 823 residues of BCL11B formed the fusion protein. The codon 20 of BCL11B was the first involved in the fusion and it encoded for an alanine instead of a proline, due to a single nucleotide substitution at the breakpoints junctions (yellow dot).

Sample #20 was also characterized by the in-frame transcript *SAV1-GYPB*, which remained cryptic at cytogenetic analysis. The driver score (DS) predicted by Pegasus (DS = 0.87) identified the chimera as a potential driver of leukemogenesis [12,13] (tier 2, Figure 2A and Figure S1B). In sample #21 we identified a novel fusion event between chromosomes 19 and 7, involving the genes *OAZ1* [14] and *MAFK* [15] (tier 2, Figure 1, Figure 2B and Figure S1C).

We validated the out-of-frame fusion *UTP6-CRLF3* [16,17] in sample #68187 (tier 3 Figure 1 and Figure S1D). *UTP6* and *CRLF3* mapped on the minus strand of chromosome 17q11 (chr17:30188190-30230729 and chr17:29107702-29153778, respectively). These genes flanked the *NF1* locus and the rearrangement suggested the presence of a 1 Mb copy number loss, which encompasses the *NF1* gene. We also confirmed the presence of the out-of-frame fusion *PUF60-TYW1* [18,19,20] in sample #125 (tier 3 Figure 1 and Figure S1E). Sample #59810 showed the *CNOT2-WT1* [2,21,22,23] chimera, which is a novel out-of-frame fusion (tier 1, Figure 1 and Figure S1F) related to t(11;12)(p15;q22) translocation, identified by cytogenetic analysis. The breakpoint mapped in the forward strand of chromosome 12 and the reverse strand of chromosome 11. We also detected a variant that mapped in exon 3 of a non-coding transcript of *CNOT2* (NR_037615). The partner genes mapped at opposite strands, the *CNOT2* and *WT1* sequence thus displayed a conserved and inverted sequence orientation, respectively.

In addition to the *CNOT2-WT1* rearrangement, sample #59810 carried the fusion transcript *ZEB2-BCL11B* [24,25,26] (tier 1, Figure 1, Figure 2C and Figure S1G), which is an in-frame fusion and a rare event in AML associated with t(2;14)(q22.3;q32.2)18. Of note, we identified three splicing isoforms (Figure S1H–I), two of which have never been reported before. The type 1 isoform was the full-length chimera that retained all exons involved in the translocation. The type 2 isoform was formed by fusion of the junction of exon 2 of *ZEB2* and exon 3 of *BCL11B*. In the type 3 isoform, exon 2 and 3 of BCL11B were removed, resulting in a smaller transcript encoded by exon 2 of *ZEB2* and exon 4 of *BCL11B*. The reciprocal fusion transcript, formed by exon 1 of *BCL11B* and exon 3 to 10 of *ZEB2*, was also detected and validated (Figure S1J). Details for each chimera are reported in Figure 1, Table 2 and Table S3.

### 2.3. Expression of Genes Involved in Fusions and Frequency of Rearrangments Across Cancers

We evaluated the expression of each gene involved in the fusions by comparing its expression to the mean expression of the same gene in wild-type patients of the cohort (Figure S3A). The genes with the most variable expression between fused and wild-type patients were *CRLF3*, *CNOT2* and *WT1*. However, due to the limited number of samples, we could not perform additional statistical analysis to test the significance of our data.

To define the transcriptional program associated with AML carrying the fusion genes, we selected the 1000 most variable genes (based on median absolute deviation values) and we performed unsupervised clustering analysis. Figure S3B showed three clusters, one of which was defined by the *ZEB2-BCL11B* rearranged case alone. The first group was characterized by the presence of the *CBFB-MYH1*, *OAZ1-MAFK* rearranged cases (sample #84 and sample #21, respectively) and a patient without fusions (sample #32). The second cluster included cases characterized by *PUF60-TYW1*, *CPD-PXT1*, *SAV1*-*GYPB* and *UTP6-CRLF3* fusions. Notably, patients carrying *CPD-PXT1* and *UTP6-CRLF3*, which were associated with *NF1* loss, clustered in this group. This cluster showed a heterogeneous transcriptional profile.

Differentially up-regulated genes (*n* = 434, logFC > 1.5) in the first cluster were enriched for genes involved in the protein processing in endoplasmic reticulum pathway, spliceosome, RNA transport and mRNA surveillance pathway (Table S4). There were no significantly down-regulated genes in the first group one compared to the second one. However, larger cohorts would be required to confirm our signature.

To collect more patients information, we downloaded data from the TCGA Tumour Fusion Gene Data Portal (https://www.tumorfusions.org/ [27]) and the Mitelman Database Chromosome Aberrations and Gene Fusions in Cancer (https://mitelmandatabase.isb-cgc.org/). We found that *ZEB2-BCL11B* (as also reported in the manuscript) and *OAZ1-MAFK* fusions were previously annotated in two AML and one multiple myeloma, respectively. Moreover, we analysed the TCGA cancer data looking for genomic rearrangements (and relative frequency) of the genes involved in the 7 fusions we detected in AML. We identified 12 genes that formed chimeras with other partners in different tumour types, namely *CPD*, *PXT1*, *SAV1*, *OAZ1*, *MAFK*, *UTP6*, *CRLF3*, *TYW1*, *CNOT2*, *WT1*, *ZEB2* and *BCL11B*. Moreover, to better understand the role of these genes in AML, we investigated their expression level in the TCGA AML cohort trough the cBio data portal (http://www.cbioportal.org/, Table S5). 

The data showed that the some of the candidate genes form chimeras with a variety of partners in different tumor types and the most frequently rearranged genes were CPD and CNOT2. On the other hand, ZEB2-BCL11B was the only recurrent fusion in acute leukemias, suggesting a pro-tumorigenic function in the hematopoietic compartment.

### 2.4. Relative Frequency of ZEB2-BCL11B Chimera in Acute Leukemia

**Figure 3:**
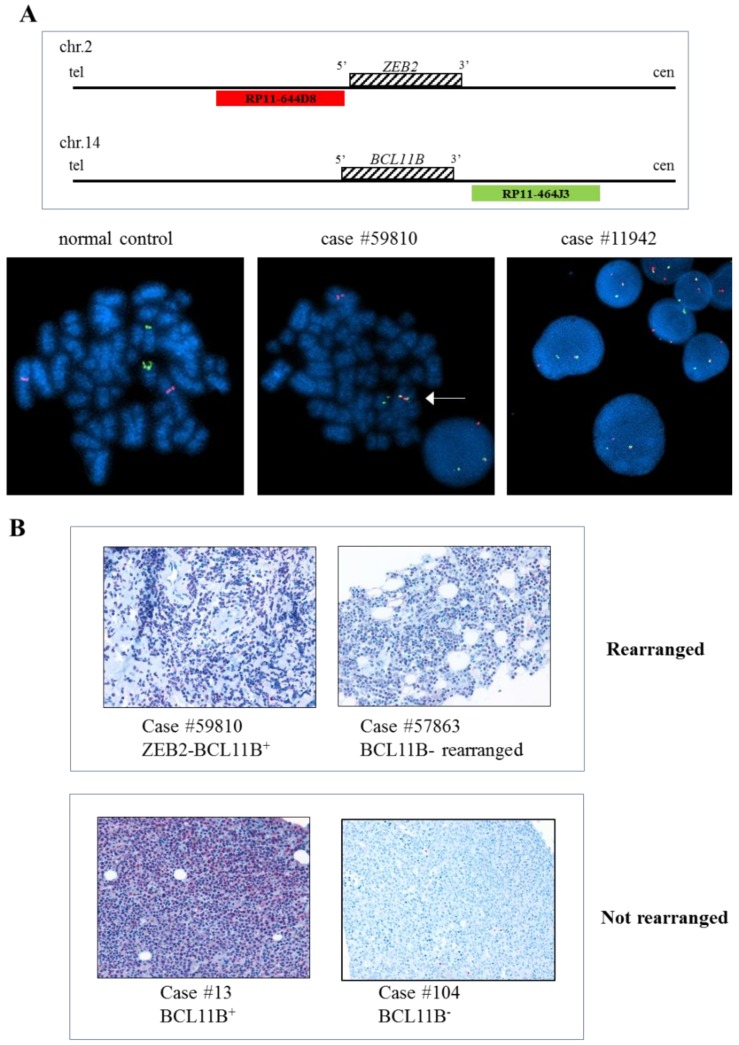
Immunohistochemistry and FISH of AML cases. (**A**) FISH analysis using specific probe for *ZEB2* and *BCL11B* flanking regions. Schematic representation of RP11-644D8 BAC probe in Spectrum Orange covering the 5’ region of *ZEB2* and RP11-464J3 BAC probe in Spectrum Green covering the 3’ region of *BCL11B*, is shown at the top. FISH performed on metaphase spread of case #59810 showing an abnormal fusion pattern (1 fusion, 1 orange and 1 green) with the fusion signal indicating *ZEB2*-*BCL11B* fusion gene on der(14) (bottom, central) and the same abnormal FISH pattern observed in interphase nuclei of case #11942 (bottom, right). A normal FISH pattern (2 red and 2 green signals) in a normal control case is shown (bottom, left). (**B**) Immunohistochemistry analysis of BCL11B-expressing AML samples #59810, #57863 and #13 carrying t(2;14)(q22.3;q32.3), t(7;14)(q21q32) and no 14q32 alteration, respectively. BCL11B expression was detected in samples regardless of the presence of the 14q32 alterations. The expression was limited to the nucleus and the percentage of positive neoplastic cells was always ≥ 50%.

The fusion protein *ZEB2-BCL11B* was previously described in AML [28] and mixed phenotype acute leukemias [29]. To investigate the frequency of the t(2;14)(q22.3;q32.2) translocation in AML, we interrogated the Mitelman Database (last update on 21 May 2018, Table S6 [30]) and found four AML cases [28,31,32,33]. Moreover, while the 14q32 region and *BCL11B* are known to be frequently altered in hematological malignancies [34], we found only three additional cases of lymphoid malignancies carrying the t(2;14)(q21;q32) translocation, including biphenotypic leukemia [35] and acute lymphoblastic leukemia [36,37,38]. However, *ZEB2* and *BCL11B* involvement was confirmed only in one case of AML present in the database. In order to extend the screening to AML patients who are potential candidates on the basis of their the cytogenetic data, we performed FISH on four additional cases carrying t(2;14)(q14-q23;q32) and we confirmed the presence of the *ZEB2-BCL11B* fusion gene in all samples (Figure 3A). Notably, the presence of the fusion was confirmed by RNA-seq in the sample #11945 [39]. At a genomic level, the breakpoint mapped at coordinates chr2:145231055-145231058 and chr14:99736728-99736731. However, it was not possible to locate the exact position of the breakpoint due to the presence of 3 cytosines in the region of the breakpoint, which could belong to either *ZEB2* or *BCL11B* (Supplementary Figure S4A). Overall, *ZEB2-BCL11B* expressing patients (*n* = 5), were characterized by a median age at diagnosis of 59 years old and by poorly differentiated morphology (Table 3). The immunophenotypic analysis was performed in three patients and two of them expressed T-cell markers. In particular, patient #11944 expressed CD2, CD7 and TdT in 94%, 82% and 26% of cells, respectively, while patient #11945 was also positive for CD3 cytoplasmatic expression, TdT and MPO, with a diagnosis of T/myeloid mixed phenotype acute leukemia (T/M MPAL). Of note, patient #11944 had a diagnosis of acute undifferentiated leukemia (AUL) and patient #59810 was positive only for myeloid markers (CD13 and CD117).

### 2.5. Specific Pattern of Mutations in Patients Carrying the ZEB2-BCL11B Chimera

We performed targeted next-generation sequencing (NGS) on a panel of genes known to be involved in myeloid malignancies to characterize the mutational landscape of patients carrying the *ZEB2-BCL11B* chimera. *FLT3* alterations were present in 4/5 (80%) patients considered (Table 4): two (40%) were characterized by the internal tandem duplication (ITD) alone with an allelic frequency > 0.5 and two (40%) had point mutations in the tyrosine kinase domain (TKD, one and two point mutations, respectively) and the ITD alteration, but with an allelic frequency <0.5 (40%). Moreover, mutations co-occurring with the *ZEB2-BCL11B* transcript and the *FLT3* alterations targeted *TET2*, *DNMT3A*, *GATA2*, *JAK2*, *RUNX1* and *SRSF2*. Notably, we did not detect any mutation of the screened genes in the patient #11942, who was also negative for *FLT3* aberrations. In addition, Immunoglobulin (IG) and T cell receptor (TCR) molecular analysis showed a clonal rearrangement in the IG heavy chain (IGH) locus, mapping at 14q32 in sample #11944 (AUL), which had a previous history of diffuse large B cell lymphoma, and a TCR rearrangement in sample #11945 (T/myeloid MPAL).

### 2.6. BCL11B Protein Expression in AML and Its Transcriptional Signature

The pro-tumorigenic role of the *ZEB2-BCL11B* fusion has been previously linked to the overexpression of *BCL11B* [28,40]. Paraffin-embedded tissue was available for one of the patients carrying the chimera (#59810) and *BCL11B* expression was confirmed at protein level by immunohistochemistry (Figure 3B).

To understand whether BCL11B expression is a more general feature of AML, we performed immunohistochemistry analysis of 21 additional cases of newly-diagnosed AML not carrying the fusion genes. We detected CD34 expression in 14/21 samples and aberrant nuclear and cytoplasmic nucleophosmin expression in 7/21 biopsies. BCL11B positivity was detected in 9/21 (40.9%) cases of AML (Table S7). BCL11B protein expression in leukemic blasts was limited to the nucleus and varied in strength from weak to moderate. Scattered cells with a stronger positivity could occasionally be seen. In positive cases, the percentage of positive neoplastic cells was always ≥50%. No significant association was found between BCL11B expression and AML immunohistochemical phenotype.

In addition, BCL11B stained positive in one T/M MPAL (CD34, MPO positive, CD3 positive) and one AUL (CD34 positive, CD117 positive and CD2 positive) corresponding to cases #193 and #57863 carrying BCL11B rearrangements and associated to t(6;14)(q25;q32) and t(7;14)(q21;q32), respectively (Table S7, Figure S4B–D).

To identify the transcriptional signature associated with BCL11B expression in AML, we studied the gene expression profile (GEP) data of patients. In our cohort (*n* = 22), 5% of AML patients had higher expression of *BCL11B* mRNA, however 10 (45.5%) and 12 (54.5%) cases either expressed BCL11B protein or did not, respectively. Of note, no significant difference was observed in terms of mRNA expression between BCL11B^+^ and BCL11B^−^ patients (mRNA data from array and qPCR, Supplementary Figure S5A), indicating the lack of association between *BCL11B* mRNA and protein levels. When comparing GEP according to protein expression, we identified 152 differentially expressed genes (*p* < 0.05), of which 36 and 116 were ≥ 2-fold upregulated and downregulated, respectively. Notably, BCL11B^+^ patients were enriched for downregulated genes involved in the innate immune response (ES = 6.3; *p* = 1 × 10^−8^), inflammatory response (ES = 5; *p* = 2.5 × 10^−5^), leukocyte migration (enrichment score ES = 9.1; *p* =1.1 × 10^−4^), cell adhesion (ES = 3.4; *p* = 0.002), leukotriene metabolic process (ES = 31.7; *p* = 0.004) and response to oxidative stress (ES = 5.8; *p* = 0.03, Table S8). Of note, among genes deregulated in the leukotriene pathway we identified *ALOX5* (fold change = −4.36) and *ALOX5AP* (fold change = −2), where the loss of *ALOX5* has been reported to impair leukemic stem cells and prevent the onset of chronic myeloid leukemia in mice [41].

### 2.7. ZEB2-BCL11B Expression Failed to Sustain Self-Renewal of Murine Hematopoietic Stem and Progenitor Cells

We assessed the leukemogenic potential of the *ZEB2-BCL11B* fusion by analyzing its ability to sustain self-renewal of murine hematopoietic progenitor cells. Bone marrow (BM) c-Kit+ cells expressing the full-length chimera were used in colony forming unit assays. In addition, cells were kept in liquid culture to monitor GFP expression and *ZEB2-BCL11B* mRNA levels over time: GFP expression increased from 2.5% at day 1–43% GFP+ cells at day 14, while mRNA levels were 30-fold and 800-fold higher than those of the negative control at day 6 and 13, respectively, highlighting low but specific expression of the chimeric transcript (Figure S5B–D). No differences in term of clonogenic capacity were detected between cells transduced with the empty vector (negative control) or the *ZEB2-BCL11B* transcript. Moreover, regarding self-renewal capacity, no colonies were detected at day 14 (second re-plating) in either the negative control or cells expressing the chimera, whereas *MLL-AF9* transduced cells (positive control) showed self-renewal capacity.

## 3. Discussion

Several studies have described a heterogeneous landscape of chimeras in AML [29,39,42,43], where very few fusions and genes were recurrently rearranged or altered. Here we analysed a cohort of AML patients characterized by the presence of a rare or never before reported chromosomal translocation with the aim of detecting the putative fusion gene correlated with the translocation. We identified novel and rare fusion events with an expected pathogenic role in adult AML patients.

The advantages of RNA-seq in detecting fusion events rely not only on the ability to systematically identify fusions whose partner genes are unknown, but also to detect those rearrangements that remain cryptic at cytogenetic analysis (small deletions, inversions or duplications). In the past years, several bioinformatics tools have been established for the detection of fusion events in RNA-seq data. However, the output of these software is represented by a high number of false positive predictions. This is mainly due to systematic errors including read-through artefacts, reverse transcriptase template switching events or mapping biases. Moreover, fusions identification tools provide no information regarding the oncogenic relevance of the output fusions. These features make the systematic experimental validation of gene fusion lists obtained from in silico pipelines unfeasible. To overcome this limitation, we exploited the “downstream” tool FuGePrior to reduce the number of events to those highly reliable and with a putative biological function. FuGePrior combines results from state of the art bioinformatic tools for chimeric transcripts identification and prioritization, several filtering and processing steps designed on up-to-date literature on gene fusions and analysis of the potential functionality of the fusion according to its structure. This allowed us to conduct the experimental validation on a manageable list of candidates.

Five fusion genes associated with the known cytogenetic translocations and four fusions that remained cryptic at the level of cytogenetic analysis were closely studied. The fusions associated with balanced rearrangements were: (i) two isoforms of *ZEB2-BCL11B* and its reciprocal *BCL11B-ZEB2* chimeric transcript associated with the translocation t(2;14)(q21-q23;q32); (ii) *CNOT2-WT1* which derived from the translocation t(11;12); (iii) *CPD-PXT1* related to the t(6;17) aberration (Figure 1 and Figure 2). Further cryptic fusions included *UTP6-CRLF3*, *PUF60-TYW1*, *SAV1-GYPB* and *OAZ1-MAFK* (Figure 1 and Figure 2). The fusions *ZEB2-BCL11B*, *BCL11B-ZEB2* and *OAZ1-MAFK* involved genes encoding for transcription factors and we speculated that the putative mechanism of action of the fusion proteins may be linked to alterations of the transcriptional program.

We selected the chimera *ZEB2-BCL11B* for functional studies due to its frequency in acute leukemia. The remaining fusion events were not further investigated. However, we speculate on their potential activity in leukemic cells according to known features of partner genes involved in the translocations.

We associated the expression of fusion events involving genes on chromosomes 17, such as *UTP6-CRLF3* and *CPD-PXT1*, to the loss of *NF1.* The detection of these “hidden” alterations required the integration of different layers of genomic data (mutation analysis and copy number alterations), highlighting the complexity of the genomic alterations in AML and the importance of an accurate characterization of each patient’s alterations to permit a personalized medicine approach. The consequences of the out of frame fusions *CNOT2-WT1* and *PUF60-TYW1* is more difficult to speculate on but may be related to the loss of function of *WT1* (data not shown) and *PUF60*, respectively. Genomic alterations of *WT1* including point mutations and small insertions and deletions have been reported in 5% of AML cases [2,43] and the haploinsufficiency of *PUF60* has been associated with the progression of T-ALL in a mouse model with homozygous deletion of *TP53* [20]. However, functional studies are needed to elucidate *PUF60* role in AML. The fusion gene *SAV1-GYPB* may be of interest due to the role of the tumor suppressor *SAV1* [44]. SAV1 interacts with two kinases MST1 and MST2 to form an active protein complex and promotes cell-cycle exit. The ability of SAV1 to binds MST1/MST2 is limited to the functionality of its coiled-coil domain. In this scenario, the identified translocation impaired the coiled-coil domain, suggesting the loss of stability of the SAV1-MST1-MT2 complex [45].

Data from the TCGA Fusion Gene Database showed that the some of the candidate genes form chimeras with a variety of partners in different tumor types, suggesting that they might locate in genomic regions prone to chromosomal rearrangements [46,47] and/or have a role in carcinogenesis. The most frequently altered genes were *CPD* and *CNOT2*, whose overexpression was associated with survival, inhibition of apoptosis and angiogenesis in different cancer types [22,48,49,50,51]. Regarding the other genes that were rarely rearranged across cancer, they might participate to the leukemic phenotype, even though not being the driver of transformation. Our AML cohort was characterized by mutations in genes with a known pathogenic role in leukemia and the identified chimeras contributed to the disease complexity, as demonstrated by the involvement of genes such as *WT1* or copy-number loss of *NF1.*

Finally, we detected three isoforms of the rare fusion transcript *ZEB2-BCL11B* (sample #59810) and its reciprocal *BCL11B-ZEB2*. Interestingly, the fusion protein ZEB2-BCL11B was previously identified in two adult AML cases [28,39] and three paediatric T/M MPAL cases [29], suggesting a putative role in leukemogenesis. We described the characterization of five cases carrying the t(2;14)(q22.3;q32.2) translocation involving the rearrangement of *ZEB2* and *BCL11B*. In two of the three patients with immunophentoypic characterization, leukemic cells co-expressed T-cells markers such as CD3, CD2 and CD7, and one additional case was diagnosed as AUL. Molecular profiling revealed that four out of five rearranged patients harboured *FLT3*-ITD internal tandem duplication, and two of these had an allelic fraction < 0.5 and carried a co-occurring alteration in the tyrosine kinase domain. These data suggested that *FLT3* alterations might arise as a secondary event. *In vitro* expression of the full-length *ZEB2-BCL11B* transcript in murine c-Kit^+^ cells did not show evidence of transforming ability. This evidence suggests that as for other fusions, additional alterations are required for malignant transformation [52,53] and, based on our data, *FLT3* alterations might be the most promising candidates. The elucidation of the mechanism(s) of leukemogenesis driven by the t(2;14)(q22.3;q32.2) translocation deserves further investigation. Recent studies have shed light on the role of *ZEB2* in normal and malignant haematopoiesis [24,25], suggesting its loss of function or aberrant function may also contribute to neoplastic transformation.

Interestingly, by immunohistochemistry we showed that BLC11B is expressed in the t(2;14)(q22.3;q32.2)-rearranged leukemic blasts (patient #59810), but also in nine non-rearranged AML cases and two T/M MPAL or AUL with 14q32 rearrangement. This suggests that *BCL11B* may have a role in leukemogenesis. The comparison of gene expression profile from BCL11B^+^ and BCL11B^-^ patients revealed downregulation of genes involved in the innate immune response, inflammatory response, leukocyte migration and cell adhesion, leukotriene metabolic pathways and response to oxidative stress in BCL11B^+^ AML patients. Abbas and colleagues showed that *BCL11B* overexpression in 32D myeloid cell line resulted in a decreased proliferation, less maturation toward granulocyte and more undifferentiated blast cells [40], but did not detect a transforming ability of *BCL11B*. Thus, further studies are needed to clarify the role of and interplay between the chimeric protein and co-occurring alterations in acute leukemia in an effort to identify potential therapeutic targets for these patients.

## 4. Materials and Methods

### 4.1. Patients and Samples

The study was approved by the Institutional Ethical Committee (protocol number 253/2013/O/Tess and 112/2014/U/Tess) of Sant’Orsola-Malpighi Polyclinic (Bologna, Italy) and the Internal Review Board of MLL Munich Leukemia Laboratory and was carried out in accordance with the ethical standards laid down in the 1964 Declaration of Helsinki. Samples from adult patients with primary adult AML were obtained after informed consent.

Leukocytes were enriched by separation on Ficoll density gradient and lysed in RLT buffer. Genomic DNA and RNA were extracted by column purification (AllPrep DNA/RNA/Protein Mini Kit and QIAcube, or RNeasy Mini Kit, Qiagen, Hilden, Germany).

### 4.2. Chromosome Banding Analysis (CBA)

CBA was performed as previously described [54]. Karyotypes were examined after GAW or GAG banding technique and described according to International System for Human Cytogenomic Nomenclature (ISCN 2016) [55].

### 4.3. Fluorescent In Situ Hybridization (FISH)

FISH analysis was carried out on fixed nuclei obtained using the CBA technique according to the manufacturer’s instructions. Dual color breakapart FISH probes created with the BAC clones RP11-644D8 and RP11-360D1 (covering up- and down-stream regions of the *ZEB2* gene) and with RP11-1147k11 and RP11-464J3 (covering the up- and down-stream regions of the *BCL11B* gene), was used to identify *ZEB2* and *BCL11B* rearrangements, respectively. To identify the specific *ZEB2*-*BCL11B* fusion gene, a dual color single fusion was obtained using RP11-644D8 and RP11-464J3 clones. BAC clones were provided already marked in Spectrum Orange or Spectrum Green (Empire Genomics, New York, NY, USA). The slides were counterstained with DAPI and analysed using fluorescent-microscopes equipped with FITC/TRITC/AQUA/DAPI filter sets and the Genikon imaging system software (Nikon Instruments, Tokyo, Japan). At least 100 nuclei were analysed for each sample.

### 4.4. Sequencing and Fusion Detection

Libraries for RNA-seq were prepared with the TruSeq stranded mRNA kit (Illumina, San Diego, CA, USA) following manufacturer’s instructions. RNA-seq libraries were subjected to 2 × 75 bp paired-end sequencing and run on a HiSeq 2500 or 1000 instrument (Illumina), and following manufacturer’s specifications. An average of 50 million reads *per* sample was obtained. Targeted DNA sequencing of myeloid-related genes was performed using the TruSight Myeloid Sequencing Panel (Illumina) and run on a MiSeq instrument (Illumina). Variants with a total read depth > 500 and falling into exonic regions and splice sites were retained. Targeted sequencing of *ZEB2-BCL11B* rearranged patients was performed as previously described [39].

Fusion genes were detected on RNA-seq data by applying FuGePrior pipeline to the gene fusion lists provided by ChimeraScan [56] and deFuse [57] tools. According to FuGePrior workflow [58], fusions with the following features were removed: (i) not supported by split reads (i.e., reads harboring the fusion breakpoint); (ii) involving at least one unannotated partner gene; (iii) shared by healthy samples; (iv) characterized by a non-reliable structure; (v) having at least the driver score probability lower than 0.7. The DS score was a measure of the probability of the fusion being an oncogenic event, according to Pegasus [59] and Oncofuse [60].

Firstly, we screened the putative fusions list to identify chimeras originating from chromosomal translocations detected by the cytogenetic analysis (tier 1). Secondly, to identify cryptic fusions and to reduce the number of false-positive predictions, we implemented additional filters to remove: (i) recurrently fused genes showing a large diversity among partner genes (including *HBB*, *HBA*, *HBD*, *MPO*, *DLG2*) [61]; (ii) conjoined genes; (iii) fusions recurring in more than one sample in our cohort. We added the latter criteria as we assumed it was not likely to found a recurrent fusion in such a small and heterogeneous cohort. Then, in order to identify cryptic but relevant fusions, we prioritized chimeras according to the probability of the transcript being an oncogenic event (tier 2). Finally, we rescued out-of-frame fusions (DS < 0.7) involving tumor suppressor genes (tier 3) to identify loss of function alterations in key genes. The recurrent gene fusion *CBFB-MYH11* was identified in the positive control (sample #84), thus confirming the reliability of our bioinformatic analysis. The dataset supporting the conclusions of this article is available in the NGS-PTL repository, at the following link: https://ngs-ptl.unibo.it:5006.

For expression analysis, raw data were aligned to the reference genome and read counts were normalized using the DESeq2 package and the rlog transformation for data normalization [62]. Differentially expressed genes, median absolute deviation calculations, unsupervised clustering and expression plots were performed using R packages limma [63], DescTools, ComplexHeatmap [64] and ggplot2, respectively. Enrichment pathway analysis was performed with Enrichr [65].

### 4.5. RT-PCR, PCR, qPCR and Sanger Sequencing

cDNA synthesis was performed using M-MLV Reverse Transcriptase for primary AML samples and Random Hexamers (Invitrogen, Thermo Fisher, Waltham, MA, USA) or the SuperScript III First-Strand Synthesis System (Invitrogen) for RNA extracted from transduced c-Kit^+^ cells. Polymerase chain reaction (PCR) primers were designed to amplify fragments containing the fusion boundary detected by RNA-seq using Primer3 (http://primer3.ut.ee/, Table S1). Quantitative PCR (qPCR) was performed using Brilliant III Ultra-Fast QPCR Master Mix (Agilent Technologies, Santa Clara, CA, USA) on an Mx3000p qPCR system (Agilent Technologies) and standard cycling set-up (Table S1). TaqMan gene expression for *BCL11B* mRNA (Hs01102259_m1) was performed on BM cells from AML patients (blasts ≥ 80%, *n* = 10) and peripheral blood mononuclear cells from healthy controls (*n* = 3), using *GAPDH* (Hs02786624_g1) as reference gene, on the Applied Biosystems 7500 Real-Time PCR System (Thermo Fischer Scientific). Gene expression was quantified by the 2^-ΔΔCt^ method, using the average of healthy controls as reference sample. Long-distance PCR were performed with LA Taq DNA Polymerase (Takara Bio, Shiga, Japan) following manufacturer instructions for human genomic DNA. Fast Start Taq DNA Polymerase (Roche, Basel, Switzerland) was used for standard PCR reactions. Products were purified with the QIAquick PCR purification kit (Qiagen) or conventional agarose gel electrophoresis and extraction of specific bands with the QIAquick Gel Extraction kit (Qiagen). PCR products were sequenced by Sanger Sequencing using an ABI PRISM 3730 automated DNA sequencer (Applied Biosystems) and the Big Dye Terminator DNA sequencing kit (Applied Biosystems, Foster City, CA, USA). Fusion detection was performed using NCBI Blast alignment and BLAT software tool (http://genome.ucsc.edu/cgi-bin/hgBlat?command=start) to reference genome GRCh37/hg19.

BCR and TCR clonality assay was performed as described by the BIOMED-2 study [66].

### 4.6. Immunohistochemistry

BM specimens were fixed in B5 solution for 2 hours, decalcified with EDTA-based solution for 3 hours and paraffin embedded. Histological stainings were examined (Hematoxylin&Eosin, Giemsa, Gomori silver impregnation) and 3 μm-thick sections were cut for immunohistochemistry. The antigen retrieval methods used were heat-based Pt-Link (Agilent Technologies, PT100/PT101) and EnVision Flex Target Retrieval Solution High pH (Agilent Technologies, K8004) at 92 °C or 82 °C. All samples were stained for the following molecules: CD34 (mouse monoclonal, clone END, NCL-L-END,1:100, Microsystems, Newcastle, UK), myeloperoxidase (rabbit polyclonal, A0398, 1:5000, Agilent Technologies), CD68 (mouse monoclonal, clone PGM1, 1:5, kindly provided by Prof. Falini, Perugia, Italy), BCL11B (rabbit polyclonal, NB100-2600, 1:200, Novus Biologicals Centennial, CO, USA). The BCL11B antibody was validated on reactive bone marrow and nodal follicular hyperplasia. The staining panels on the AML cases were performed using positive (the same sample for validation) and negative controls (slides with exclusion of the primary antibody). The analysis of CD34 and CD68 antibodies were performed according to long standing previously settled procedures.

The reaction detection was performed by using the Dako Real Detection Systems Alkaline Phosphatase/RED Rabbit/Mouse Kit (K 5005, Agilent Technologies). Overall, 24 BM biopsies were analysed. One BM biopsy referred to case #59810 with t(2;14), 21 BM biopsies referred to 21 AML patients without t(2;1) and/or 14q32 rearrangement, 2 BM biopsies referred to patients with 14q32 rearrangements (Table S6).

### 4.7. Gene Expression Profiling (GEP) and SNP-Array

We analysed gene expression and copy number data from a previously obtained internal cohort [54]. Gene expression raw data were processed by Expression Console software with Signal Space Transformation Robust Multi-Array average (sst-RMA) normalization. Supervised data analysis was carried out with Transcriptome Analysis Console v4.0 software (Affymetrix, Thermo Fisher). Functional annotation clustering and enrichment analysis was performed using David Bioinformatics Resources 6.8 (National Institute of Allergy and Infectious Diseases, NIH) [67]. CEL files from SNP-array raw intensities were processed using Rawcopy [68].

### 4.8. Retroviral Transduction Assays

The TY1-tagged full length transcripts *ZEB2-BCL11B* was subcloned into a retroviral vector using EcoRI restriction sites. The resulting plasmid’s sequence was verified by Sanger sequencing. Murine stem cell virus–based (MSCV-based) retroviral constructs carrying the tagged *ZEB2-BCL11B* sequence upstream of an internal ribosomal entry site–green fluorescent protein (IRES-GFP) cassette were generated using 293T packaging cell line. Vectors containing the fusion gene (*ZEB2-BCL11B*), the *MLL-AF9* fusion (acting as positive control) or the empty vector (negative control) were used to transduce mouse c-Kit^+^ BM cells. Mouse whole BM was positively selected with the CD117 (c-Kit) MicroBeads and the LS MACS column according manufacturer’s instructions (Miltenyi Biotec, Bergisch Gladbach, Germany). Retroviral transduction was performed as previously described [69].

### 4.9. Serial Colony Replating Assay

Colony forming unit assay was performed in duplicates by seeding 1000 c-Kit^+^ transduced cells in Methocult M3434 methylcellulose medium (StemCell Technologies, Vancouver, BC, Canada). Cells were plated in duplicate and after 7–12 days colonies were scored, pooled and identical numbers of cells were re-plated under the same conditions.

### 4.10. Flow Cytometry Analysis

Multiparameter flow cytometry (MFC) and sample processing was carried out as described previously [70]. MFC analyses were performed using FC500 or Navios flow cytometers (Beckman Coulter, Miami, FL, USA). List mode files were analyzed using CXP Software version 2.0 and Kaluza version 1.0 (Beckman Coulter, Brea, CA, USA). Diagnoses were assigned according to EGIL and WHO classifications [10,71]. Single cell suspensions of transduced c-Kit^+^ cells were prepared as described elsewehere [15]. Dead cells were excluded by gating on 7AAD (Miltenyi Biotec)-negative cells. Flow cytometry analysis were performed on an LSR Fortessa cell analyser (BD Biosciences, San Jose, CA, USA) and data were analysed with FlowJo software v 10 (BD, Franklin Lakes, NJ, USA).

### 4.11. Immunoblotting

Whole-cell lysates were prepared from 10^7^ cells in 6× Laemmli buffer. Lysates were run on SDS–PAGE gels and transferred to PVDF membranes (Millipore). Membranes were probed with the anti-Gapdh (Abcam, Cambridge, UK), anti-TY1 (Thermo Fisher Scientific) and anti-BCL11B (Abcam) primary antibodies at 1:10000, 1:2000 and 1:10000 dilutions, respectively. Membranes were probed with secondary antibodies conjugated to IRDye 680RD or IRDye 800 CW (LI-COR Biosciences Ltd. Lincoln, NE, USA) at 1:10000 dilution and proteins were detected using the Odyssey Infrared Imaging System (LI-COR Biosciences Ltd). Restore Western Blot Stripping Buffer (Thermo Fisher Scientific) were used to remove primary and secondary antibodies from PVDF membrane in order to reprobe with the anti-BCL11B antibody.

## 5. Conclusions

Fusion genes are frequently detected in cancer and they are often the result of chromosomal rearrangements such as translocations, inversions and deletions, all of which may involve a single chromosome or different chromosomes. Here we reported the identification of novel gene fusion events in AML. Although the pathogenic role and functional properties of these alterations will require additional functional studies, here we demonstrated that *ZEB2-BCL11B* rearrangement is recurrent and associated with distinct immune-clinico characteristics.

## Figures and Tables

**Table 1:** Patient characteristics and number of validated fusions per patient.

ID	Karyotype Main Clone	Karyotype Second Clone	Karyotype Other Clones	Blasts	WHO Classification	Other Genetic Abnormalities	Phase	Validated fusion(s)
59810	46,XX,t(2;14)(q21;q32),t(11;12)(p15;q22) [17]	46,XX [3]	NA	80%	AML NOS, without maturation	*FLT3*, *TET2*	Diagnosis	2
20	46,XY,t(6;17)(p21;q11) [20]	NA	NA	90%	AML NOS, with maturation	*NRAS*, *SRSF2*, *STAG2*, *TET2*	Diagnosis	2
21	46,XY,t(3;12)(p22;q24),+4,-15,+mar [19]	46,XY [1]	NA	80%	AML with mutated *RUNX1* (provisional entity)	*CBL*, *DNMT3A*, *IDH2*, *KDM6A*, *RUNX1*	Relapse	1
32	45,XY,der(12)t(12;18)(p13;q12),-18 [12]	45,XY,t(4;16)(q31;q22),der(12)t(12;18)(p13;q12),-18 [4]	45,XY,der(6)t(6;12;18)(p21;p13,q12),-18 [3]/46,XY [1]	80%	AML with MRC	*FLT3*, *WT1*	Relapse	0
84	47,XX,+8,del(11)(p11p15),t(15;17)(q24q25), inv(16)(p13q22) [20]	NA	NA	80%	AML with inv(16)(p13.1q22)	*CUX1*, *NOTCH1*	Diagnosis	1
68187	46,XX,add(8)(p23),der(16)t(1;16)(q11;q11) [18]	46,XX [2]	NA	70%	AML with MRC	*ETV6*, *KDM6A*	Diagnosis	1
63569	46,XY [20]	46,XY,add(10)(p15) [9]	46,XY,add(10)(p15),t(1;8)(p36;q13) [2]	70%	AML with mutated *NPM1*	*DNMT3A*, *FLT3*, *IDH2*, *NPM1*	Relaspe	0
125	46,XX [11]	44~47,XX,t(4;17)(p15;q21),del(5)(q13q33),-7,-18,der(X),+1~3mar [9]	NA	50%	AML with MRC	*TP53*	Diagnosis	1

Sample #84: positive control; NOS = not otherwise specified; MRC = myelodysplasia-related changes. NA = not available. Numbers in squared brackets indicates the number of cells with the relative karyotype.

**Table 2:** Biological function of genes affected by a fusion event and their potential role in leukemogenesis.

Sample	Fusion	Gene Function	Category	Fusion Protein Putative Function
20	*CPD-PXT1*	*CPD* encodes for a metallocarboxypeptidase [11]	*NF1* loss	The breakpoint in *CPD* was associated with a complex rearrangements that involved the loss of *NF1*. The sample was also characterized by a mutation in *NF1* detected by WES.
		The role of *PXT1* is unknown		
20	*SAV1-GYPB*	SAV1 is a tumor suppressor of the Hippo pathway [12]	Tumor suppressor	Loss of function of SAV1.
		GYBP is a sialoglycoproteins of the human erythrocyte membrane [13]		
21	*OAZ-MAFK*	*OAZ1* is an Ornithine decarboxylase (*ODC*) antizyme protein that negatively regulates *ODC* activity [14]	Transcription factor	The chimera may alter the cellular transcriptional program.
		*MAFK* is a transcriptional regulator with bZIP domains [15]		
68187	*UTP6-CRLF3*	*UTP6* is involved in nucleolar processing of pre-18S ribosomal RNA and centriole duplication [16]	*NF1* loss	The rearrangement led to a CN loss involving *NF1*, which maps in the forward strand of chromosome 17: 29421945-29709134 (GRCh37).
		*CRLF3* is a cytokine receptor-like factor that may negatively regulate cell cycle progression at the G0/G1 phase [17]		
125	*PUF60-TYW1*	*PUF60* participates in the splicing machinery [18,20]	Tumor suppressor	*PUF60* haploinsufficiency was involved in *TP53*-dependent progression of a T-cell acute lymphoblastic leukaemia [20].
		*TYW1* may be a component of the wybutosine biosynthesis pathway [19]		
59810	*CNOT2-WT1*	*CNOT2* encodes for a subunit of the multi-component CCR4-NOT complex, which is involved in transcriptional regulation and mRNA degradation [21,22,23]	Tumor suppressor	The translocation was associated to a deletion at 5’ of *WT1,* which lead to its CN loss.
		*WT1* is a transcription factor and it is recurrently altered in haematological malignancies, including AML [2]		
59810	*ZEB2-BCL11B* and *BCL11B-ZEB2*	*ZEB2* is a transcriptional factor involved in normal and malignant haematopoiesis [24,25]	Transcription factor	The chimera may activate an aberrant transcriptional programme.
		*BCL11B* is a transcription factor and key regulator of both differentiation and survival of T-lymphocytes during thymocyte development [26]		

**Table 3:** Characteristics of patients carrying the *ZEB2-BCL11B* rearrangement and confirmed by FISH.

Case Number	Gender	Age	WHO Classification	Karyotype	FISH	T-cell Markers	BCR	TCR
11942	male	58	AML NOS	46,XY,t(2;14)(q23;q32)	POSITIVE	NA	no clonality detected	no clonality detected
11954	male	85	AML with mutated *RUNX1* (provisional entity)	46,XY,t(2;14)(q14;q32)	POSITIVE	NA	no clonality detected	no clonality detected
11944	male	79	AUL	46,XY,t(2;14)(q21;q32)	POSITIVE	CD2+; CD7+; TdT+	clonal	no clonality detected
11945	male	59	T/myeloid MPAL	46,XY,t(2;14)(q22;q32)	POSITIVE	CD3+; CD7+; CD2+; TdT+	no clonality detected	clonal
59810	female	40	AML NOS, without maturation	46,XX,t(2;14)(q21;q32),t(11;12)(p15;q22)	POSITIVE	negative	no clonality detected	no clonality detected

**Table 4:** Mutational status of myeloid-related genes screened by NGS.

**A**												
	**ASXL1**	**BCOR**	**CALR**		**CBL**	**CSF3R**	**CSNK1A1**	**DNMT3A**	**ETNK1**	**ETV6**	**EZH2**	**FLT3-TKD**
#11942	NEG	NEG	NEG		NEG	NEG	NEG	NEG	NEG	NEG	NEG	NEG
#11944	NEG	NEG	NEG		NEG	NEG	NEG	NEG	NEG	NEG	NEG	NEG
#11954	NEG	NA	NA		NA	NA	NA	NEG	NA	NA	NA	NEG
#11945	NEG	NEG	NEG		NEG	NEG	NEG	POS	NEG	NEG	NEG	POS
#59810	NEG	NEG	NEG		NEG	NEG	NEG	NEG	NEG	NEG	NEG	POS
**B**												
**FLT3-TKD mutation and VAF**						**FLT3-ITD**	**FLT3-ITD VAF**	**GATA1**	**GATA2**	**IDH1**	**IDH2**	**JAK2**
						NEG		NEG	NEG	NEG	NEG	NEG
						POS	>0,5	NEG	NEG	NEG	NEG	NEG
						POS	>0,5	NA	NA	NEG	NEG	POS
c.2516A>G, c.2503G>T; p.Asp839Gly, p.Asp835Tyr; 4%, 8%						POS	<0,5	NEG	POS	NEG	NEG	NEG
c.2516A>G, p.Asp839Gly 34%						POS	<0,5	NEG	NEG	NEG	NEG	NEG
**C**												
**KIT**	**KRAS**	**MPL**	**NPM1**	**NRAS**	**PHF6**	**PTPN11**	**RUNX1**	**SETBP1**	**SF3B1**	**SRSF2**	**STAG2**	**STAT3**
NEG	NEG	NEG	NEG	NEG	NEG	NEG	NEG	NEG	NEG	NEG	NEG	NEG
NEG	NEG	NEG	NEG	NEG	NEG	NEG	VARIANTE	VARIANTE	NEG	POS	NA	NEG
NA	NEG	NA	NEG	NEG	NA	NA	POS	NA	NEG	NA	NA	NA
NEG	NEG	NEG	NEG	NEG	NEG	NEG	VARIANTE	NEG	NEG	NEG	NEG	NA
**NEG**	NEG	NEG	NEG	NEG	NEG	NEG	NEG	NEG	NEG	NEG	NEG	NEG
**D**												
**STAT5B**		**TET2**		**TP53**		**U2AF1**	**WT1**	**ZRSR2**				
NEG		VARIANTE		NEG		NEG	NEG	NEG				
NEG		POS		NEG		NEG	NEG	NEG				
NA		NEG		NEG		NA	NA	NA				
NA		NEG		NEG		NEG	NEG	NEG				
NEG		POS		NEG		NEG	NEG	NEG				

NEG: negative; POS: positive; VAF: variant allele frequency; NA: data not available.

## Supplementary Materials

The following are available online at https://www.mdpi.com/2072-6694/11/12/1951/s1, Table S1. Primers used for validation by RT-PCR and Sanger sequencing. Table S2. Mutational status of myeloid-related genes screened by NGS. Table S3. List of 19 fusions selected for validation with RT-PCR and Sanger Sequencing and their relative annotation, driver scores (according to Pegasus and Oncofuse), tier and cDNA breakpoints for validated chimera. Table S4. Enrichment pathway analysis of differentially expressed genes among groups identified by unsupervised clustering. Table S5. List of annotated cases in the TCGA Fusion Portal and their expression level as reported in the TCGA AML cohort in the cBio data portal. Table S6. List of annotated cases in the Mitelman database affected by haematological malignancies and characterized by the presence of a translocation between chromosome 2q21-23 and the 14q32 region. Figure S1. Electropherogram of fusion junctions. Figure S2. Genomic localization of copy number loss linked to the *CPD-PXT1* fusion. Figure S3. Expression analysis. Figure S4. Characterization of 14q32 genomic breakpoint A. Sequence and chromatogram of the genomic breakpoint. Figure S5. Expression of *BCL11B* mRNA and *ZEB2-BCL11B* in transduced cells.
